# Bisphenol A-associated epigenomic changes in prepubescent girls: a cross-sectional study in Gharbiah, Egypt

**DOI:** 10.1186/1476-069X-12-33

**Published:** 2013-04-16

**Authors:** Jung H Kim, Laura S Rozek, Amr S Soliman, Maureen A Sartor, Ahmed Hablas, Ibrahim A Seifeldin, Justin A Colacino, Caren Weinhouse, Muna S Nahar, Dana C Dolinoy

**Affiliations:** 1 Department of Environmental Health Sciences, School of Public Health, University of Michigan, Ann Arbor, MI, USA; 2 Department of Otolaryngology, University of Michigan, Ann Arbor, MI, USA; 3 Department of Epidemiology, University of Nebraska Medical Center, Nebraska, USA; 4 Center for Computational Medicine and Bioinformatics, University of Michigan, Ann Arbor, MI, USA; 5 Tanta Cancer Center and the Gharbiah Cancer Society, Tanta, Egypt

**Keywords:** Bead array, Bisphenol A, Egypt, Epigenetics, DNA methylation

## Abstract

**Background:**

There is now compelling evidence that epigenetic modifications link adult disease susceptibility to environmental exposures during specific life stages, including pre-pubertal development. Animal studies indicate that bisphenol A (BPA), the monomer used in epoxy resins and polycarbonate plastics, may impact health through epigenetic mechanisms, and epidemiological data associate BPA levels with metabolic disorders, behavior changes, and reproductive effects. Thus, we conducted an environmental epidemiology study of BPA exposure and CpG methylation in pre-adolescent girls from Gharbiah, Egypt hypothesizing that methylation profiles exhibit exposure-dependent trends.

**Methods:**

Urinary concentrations of total (free plus conjugated) species of BPA in spot samples were quantified for 60 girls aged 10 to 13. Genome-wide CpG methylation was concurrently measured in bisulfite-converted saliva DNA using the Infinium HumanMethylation27 BeadChip (N = 46). CpG sites from four candidate genes were validated via quantitative bisulfite pyrosequencing.

**Results:**

CpG methylation varied widely among girls, and higher urinary BPA concentrations were generally associated with less genomic methylation. Based on pathway analyses, genes exhibiting reduced methylation with increasing urinary BPA were involved in immune function, transport activity, metabolism, and caspase activity. In particular, hypomethylation of CpG targets on chromosome X was associated with higher urinary BPA. Using the Comparative Toxicogenomics Database, we identified a number of candidate genes in our sample that previously have been associated with BPA-related expression change.

**Conclusions:**

These data indicate that BPA may affect human health through specific epigenomic modification of genes in relevant pathways. Thus, epigenetic epidemiology holds promise for the identification of biomarkers from previous exposures and the development of epigenetic-based diagnostic strategies.

## Background

The developmental origins of health and disease (DOHaD) hypothesis postulates that environmental and nutritional factors influence developmental plasticity during critical periods of development, thereby altering susceptibility to diseases later in life [1-3]. Increasingly, this emerging field of research points to the epigenome as an important modifier of disease susceptibility. Although the epigenome is vulnerable to environmental perturbations during multiple points in life, it is particularly susceptible during embryogenesis, neonatal development, and adolescence. For example, similar to the rapid synthetic rate of DNA in early development, during the pre-pubertal period, mammary growth is highly proliferative and tightly regulated [4-8], making pre-adolescent females especially vulnerable to environmental insults acting via an epigenetic mechanism.

Accumulating work suggests that exposure to endocrine-active compounds (EACs), including bisphenol A (BPA), increases susceptibility for adverse phenotypic outcomes via epigenetic mechanisms. Methylation studies conducted by Li *et al.* on the estrogenic pharmaceutical agent diethylstilbestrol (DES) observed hypomethylation in two critical DNA control regions in mice exposed *in utero* or in the perinatal period [9,10]. Exposure to the anti-androgenic fungicide vinclozolin, the estrogenic pesticide methoxychlor, or a mixture of plastic compounds during reprogramming of the germ line has been linked with epigenetic alterations that are inherited transgenerationally even in the absence of continued exposure [11-15]. Further, BPA, a high-production volume monomer used in the manufacture of polycarbonate plastic and epoxy resin, has been associated with epigenetic alterations following developmental exposure [16-19]. Using a rat model, Ho *et al.* observed multiple changes in gene-specific DNA methylation patterns in the adult male prostate, including hypomethylation of the *phosphodiesterase type 4 variant 4* (*Pde4d4*) gene following neonatal exposure to low BPA concentration (10 μg/kg of body weight BPA) [16]. Using the *A*^*vy*^ mouse model, we have shown that maternal dietary exposure to physiologically relevant concentrations of BPA results in altered DNA methylation at two metastable loci [17,19]. Moreover, restoration of normal methylation patterns occurs with maternal supplementation of genistein or methyl donors including folate, choline, betaine, and vitamin B_12_[17,20].

Recent toxicological and epidemiological data warrant the identification of epigenetic biomarkers associated with BPA exposure. For example, rodent studies have associated environmentally relevant pre- or perinatal BPA exposure with higher body weight, increased breast and prostate cancer susceptibility, altered reproductive function, and other chronic health effects [21]. In 2008, the first comprehensive human epidemiological study associated higher urinary BPA concentrations with increased risk for cardiovascular disease, type 2 diabetes, and liver enzyme abnormalities [22], and recent data correlated urinary BPA concentrations and obesity in U.S. children [23]. An analysis of 2003–04 National Health and Nutrition Examination Survey (NHANES) data found that the half life of BPA in the human body is longer than would be expected from acute exposure studies, suggesting that BPA either bioaccumulates in the body or BPA exposure occurs through non-dietary sources or both [24]. Hence, it is now hypothesized that life course exposure to environmental pollutants, such as BPA, are linked to adult disease, such as breast cancer [25].

Building upon ongoing epidemiological and exposure studies in Gharbiah, Egypt [26-30], we correlated urinary concentrations of total (free plus conjugated) species of BPA in spot samples with genome-wide CpG methylation levels in concurrently collected saliva DNA samples. We conducted an environmental epidemiology study of BPA exposure and CpG methylation in pre-adolescent girls hypothesizing that methylation profiles exhibit exposure-dependent trends.

Utilizing a semi-unbiased, quantitative genome-wide methylation array, which evaluates 27,543 individual CpG sites targeting 14,475 genes, we identified 57 agnostic and 35 biologically prioritized candidate CpG sites as well as gene pathways differentially methylated with respect to BPA concentrations. Four candidate genes were further validated using bisulfite pyrosequencing, a sensitive and quantitative method of DNA methylation detection. Using the Comparative Toxicogenomics Database (CTD) [31-33], we identified previously published candidate genes with up-regulated expression associated with BPA and found that many of the targets had BPA-associated hypomethylation in our sample set, and vice versa.

## Methods

### Study population

This study originated from a pilot study addressing environmental exposures in a developing country, and our study population was previously described in Nahar *et al.*[27]. Briefly we recruited healthy females 10–13 years of age living in either rural (N = 30) or urban (N = 30) areas of the Gharbiah province of Egypt, located 90 kilometers north of Cairo. We obtained written informed consent from the mother of each participant, and approval from the Institutional Review Boards of the University of Michigan and the Gharbiah Cancer Society were obtained before starting the study. The involvement of the Centers for Disease Control and Prevention (CDC) laboratory was limited and determined not to constitute engagement in human subject research. The average age of menarche in Egypt is 12.44 ± 1.3, and all study participants were pre-menstrual, as ascertained by asking the each mother about any prior menstrual cycles of her daughter [34]. To reduce variability arising from various exposures throughout the day, the participants provided one spot urine sample during the time period between noon to 4 pm and were measured for height, weight, waist, and hip circumference by trained nurses, using standardized techniques as previously described and validated [35,36]. In brief, each anthropometric measurement was taken twice. If the difference in the height measurements was > 0.5 centimeters (cm); if the difference in weight was > 0.2 kilograms; if the difference in waist circumference was > 0.5 cm; or if the difference in hip circumference was > 0.5 cm, then a third measurement was taken. We discarded the most discrepant value and averaged the other two. The Division of Laboratory Sciences of the National Center for Environmental Health at the CDC determined the urinary concentrations of total (free plus conjugated species) BPA as previously described [27,37]. An interviewer administered a lifestyle and diet questionnaire in Arabic in order to assess potential routes of BPA exposure, and field-collected anthropometric measurements were acquired. The questionnaire, entitled “Comparison of Xenoestrogen Levels Among Prepubertal Females in Urban and Rural Gharbiah, Egypt,” contained questions addressing residential history, personal care product usage, family history of cancer, use of canned foods, breast feeding status, and food preparation and storage behaviors.

Saliva DNA was concurrently collected using the Oragene DNA Self-Collection Kit (DNA Genotek, Ontario, Canada). The Oragene kit allows for long-term DNA storage at room temperature with little or no DNA degradation. Total genomic DNA was isolated from saliva samples using Oragene-DNA Self-Collecting kit standard protocols. Bisulfite conversion was carried out on 600 ng of genomic DNA using the manufacturer’s protocol (EZ DNA Methylation-Direct Kit, Zymo Research Corp., Orange, CA) for genome-wide methylation assessment using the Illumina Infinium HumanMethylation27 BeadChip platform (Illumina, San Diego, CA). For pyrosequencing validation 600 ng of DNA was bisulfite converted using the Qiagen EpiTect Bisulfite Conversion kit (Qiagen, Valencia, CA).

### Genome-wide DNA methylation discovery

Genome-wide DNA methylation was assessed with the Infinium HumanMethylation27 BeadChip (Illumina) performed at the University of Michigan DNA Sequencing Core facility in accordance with manufacturer’s instructions as previously published [38]. Samples exhibiting various BPA concentrations (ranging from non-detect to 12 ng/mL) were randomly applied to each of the 12-sample arrays to avoid bias by batch effect. The Illumina BeadStudio Software provided estimates, termed beta values or β-scores, of the percent methylation for each probe site based on the Cy3 and Cy5 intensities. We utilized a previously described normalization strategy that improves the correlation among replicate samples and tends to increase the level of significance when the methylation percentages are compared between groups [39].

In order to maximize power to detect methylation differences specific to higher BPA concentrations, we performed analysis both categorizing the samples into three groups: BPA-low (n = 22), BPA-intermediate (n = 14), and BPA-high (n = 10) and treating BPA as a continuous variable. First, in the categorization strategy, samples were classified as BPA-low if the BPA measurement was less than 1 ng/mL; BPA-high if BPA measurement was greater than 2 ng/mL; and BPA-intermediate for remaining samples. The observed BPA concentrations within our Egyptian cohort were relatively low, ranging from below the limit of detection (LOD) to 12 ng/mL. To identify differential methylation associated with high BPA concentrations, we performed the categorical analysis focusing on the tails of the BPA distribution. We used an empirical Bayesian moderated *t*-test to assess significance between the percent methylation in BPA-high versus BPA-low samples, which improves results for studies of small sample size [40]. Those with p-value < 0.05 with average ß-score difference greater than 5% were reported as candidate sites. To identify differential methylation associated with the highest concentrations of BPA in this cohort, the analysis described above was repeated using a more stringent cutoff for the BPA-high group (n = 5, where urinary BPA concentrations are greater than 2.60 ng/mL, the median concentration of age and gender matched individuals from NHANES 2009–2010) (Additional file 1: Table S1). The list of significant CpG sites at candidate genes from the above analysis was compared with BPA-interacting genes acquired from CTD and used for the pathway enrichment analysis.

### Bioinformatics analyses

All statistical analyses were performed with R Statistical Software (version 2.10.1). CpG sites that failed on 10% of samples were not included in subsequent analyses. Using the LIMMA package (Version 3.4.4) in R, BPA-dependent differential methylation regions among Egyptian girls were examined using covariates including hybridization date, age, breast-fed status, body mass index (BMI), and specific gravity to adjust for urinary dilutions, and log-transformed urinary concentrations of BPA. As is standard in microarray analyses, empirical Bayesian variance methods were incorporated to perform site-specific moderated t-tests [40]. The linear model used for each individual CpG site was as follows:

$$\begin{array}{l} \%\;\mathrm{Methylation}=\beta_{0}+\mathrm{breast–fed}\;\mathrm{status}\\ \;+\mathrm{hybridization}\;\mathrm{date}\\ \;+\beta_{1}\left(\log\left(\mathrm{BPA}\right)\right)+\beta_{2}\left(\mathrm{age}\right)\\ \;+\beta_{3}\left(\mathrm{BMI}\right)\\ \;+\beta_{4}\left(\mathrm{urinary}\;\mathrm{correction}\right) \end{array}$$

#### RPMM clustering analysis

The Recursively Partitioned Mixture Model (RPMM) clustering analysis in R software program was performed on the top 200 most variable CpG sites using the glcTree function, which performs Gaussian latent class modeling. The only non-default parameter used during the analysis was for the level of verbosity, which was set at 0 (default is at 2). The glcTree clustering analysis separated 46 samples into 3 leaf classes (clusters 1, 2, and 3).

#### Logistic regression-based pathway enrichment analysis

An enrichment analysis was performed using logistic regression-based pathway enrichment analysis tool (LRpath) available at http://lrpath.ncibi.org[41,42]. The data representing 27,543 sites generated from the R statistical analysis described above was reformatted to contain Entrez gene IDs, p-values, and fold-changes in tab-delimited text file format. Gene Ontology (GO) terms and pathway concept types were selected (Biocarta pathway, Edinburgh Human Metabolic Network (EHMN) metabolic pathway, GO biological process, cellular component, and molecular function, Kyoto Encyclopedia of Genes and Genomes (KEGG) pathway, and Panther pathway) for enrichment analysis in LRpath web-application. For both dichotomous comparison of low- versus high-BPA sample groups and the continuous linear models, LRpath was performed using the directional option with default settings, to distinguish pathways enriched with hyper- versus hypo-methylated sites.

#### Gene set enrichment analysis

Positional gene set enrichment analysis was performed using Gene Set Enrichment Analysis (GSEA) to determine statistical over-representation of BPA-concentration specific epigenetic marks within chromosomal cytogenic bands. The differences in average β-score of each probe (N = 27,543) between BPA-low and BPA-high groups were pre-ranked and subjected to GseaPreranked analysis tool. After collapsing features into gene symbols, a total of 14,474 genes were represented, and the enrichment among 240 cytogenic band sets were tested with default setting.

### Comparative toxicogenomics database (CTD)

The publicly available Comparative Toxicogenomics Database (CTD) combines manually curated information from peer-reviewed articles on environmental chemicals, interacting genes, and known associated health effects in human as well as in animal models [31-33]. The tool allows investigators to quickly query their epigenomics-derived gene list with curated genes associated with a particular exposure or phenotypic endpoint of interest. A list of 903 BPA-interacting genes in humans was acquired using a chemical-gene interaction query using Bisphenol-A as a search term, and after mapping the list to the genes present on the BeadChip, 657 unique genes represented by 1,355 probes remained for downstream analysis.

### Validation by pyrosequencing

CpG methylation at specific loci of the biologically-prioritized and agnostic candidates was validated by pyrosequencing using 2 ul bisulfite converted DNA. PCR and sequencing primers were designed in-house using PyroMark software are listed in Additional file 1: Table S2. The PCR products were run on a 1.5% agarose gel to ensure PCR quality, correct product length, and lack of contamination. Pyrosequencing was performed following the manufacturer’s protocol on a PyroMark MD (Qiagen).

## Results

### Characterization of DNA methylation across Egyptian girls

High-throughput analysis of DNA methylation in a cohort of Egyptian girls was performed using the Infinium HumanMethylation27 BeadChip (Illumina) containing 27,543 CpG sites targeting 14,475 gene promoters, including > 200 cancer-related and imprinted genes and 110 microRNAs [43-45]. To demonstrate experimental consistency in our research setting, we selected four samples with the most abundant genomic DNA and profiled them on the bead array in duplicate. All duplicates were shown to be highly correlated with a median correlation coefficient value of 0.98 (data not shown).

**Figure 1:**
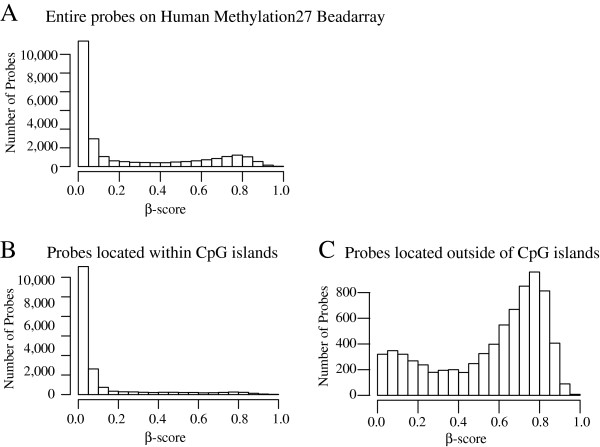
**β-score distribution in the HumanMethylation27 BeadChip in the presence and absence of CpG islands in 1 randomly chosen sample.** All samples display similar distributions as shown above. (**A**) The first histogram represents the β-score frequency of all probes. (**B**) The β-score frequency of the probes located within CpG islands is graphed. (**C**) The β-score frequency of the probes located outside of CpG islands is graphed.

We evaluated the overall distribution of the β-score, which indicates the level of methylation at CpG sites (Figure 1A). Among the 27,543 probes present in all 46 samples with a significant p-value (p < 0.05), 19,985 (73%) probes were located within annotated CpG islands, while the remaining 7,558 (27%) probes were not. More than half of the probes (average n = 11,700 with s.d. of 1,786) located within CpG islands had a β-score less than 0.05 showing highly shifted distribution (Figure 1B). Probes located outside of CpG islands displayed skewed bimodal distributions of β-scores (Figure 1C). The secondary peak observed on the long right tail of the distribution in Figure 1A is likely associated with probes from CpG island depleted regions.

### Urinary BPA concentration affects CpG methylation profiles

**Figure 2:**
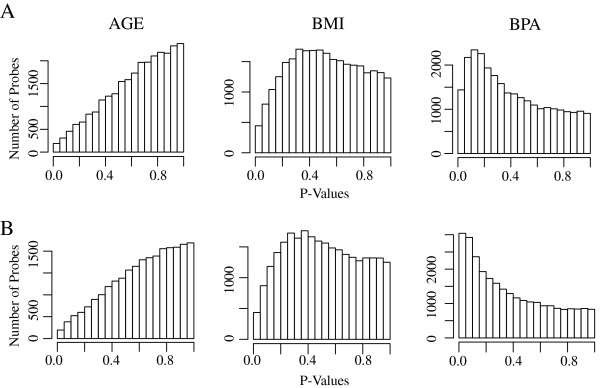
Histogram of p-values (bin = 0.1) in methylation analysis using covariates including age, BMI, and log BPA in empirical Bayes modeling using LIMMA package in R software with BPA as a (A) dichotomous or (B) continuous variable.

As described previously, urinary BPA concentrations and variability were low in the Egyptian girls cohort compared to age-matched females in the United States as reported in 2009–2010 National Health and Nutrition Examination Survey (NHANES) [27]. Concentrations of urinary BPA in Egyptian girls ranged from below the limit of detection (LOD = 0.4 ng/ml) to 12 ng/mL (total median unadjusted: 0.70 ng/mL) compared to age-matched American girls ranging from below the LOD to 16.1 ng/mL (median unadjusted: 2.60 ng/mL). Comparing samples with BPA-low and BPA-high concentrations as a dichotomous trait resulted in 1,439 differentially methylated sites (Figure 2A) with p-value less than 0.05 and 171 sites with a p-value less than 0.01, compared to 190 differentially methylated sites by age and 444 differentially methylated sites by BMI. The overall difference in average β-score between the two BPA exposure groups was not significant (0.177 vs. 0.180); however, when we limited the analysis to those sites that are differentially methylated, there were a greater number of hypo- (178 with at least 5% change in β-score) compared to hyper-methylated (54 with at least 5% change in β-score) sites in the BPA-high group compared to the BPA-low group (Additional file 2: Figure S1). The top 57 agnostic candidate genes with average β-score difference greater than 5% (ranges from −0.11 to 0.12) and a p-value < 0.05 are listed in Additional file 1: Table S3.

**Figure 3:**
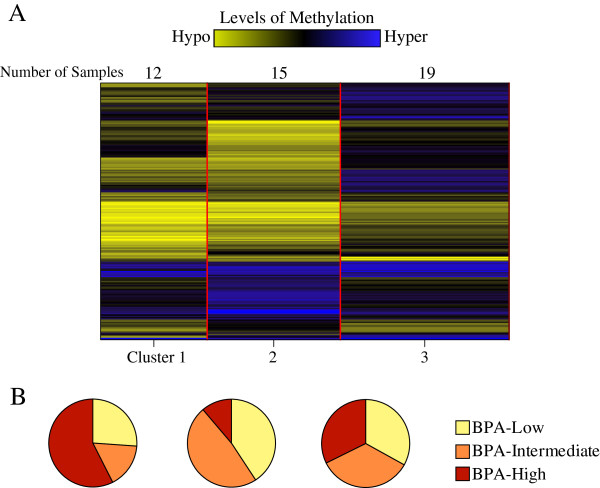
**The 200 most variable CpG sites from 46 samples were clustered using Recursively Partitioned Mixture Model (RPMM) for Beta and Gaussian Mixtures from R package, a model-based clustering algorithm that returns a hierarchy of classes, similar to hierarchical clustering and finite mixture models.** (**A**) RPMM clustering divided 46 samples into 3 clusters (1, 2, and 3), containing 12, 15, and 19 samples, respectively. (**B**) More than half of the BPA-high samples were clustered within cluster 1, while BPA-low and –intermediate samples were more evenly distributed across all 3 clusters.

Using Recursively Partitioned Mixture Model (RPMM) clustering analysis within the R software program, we examined the patterns of differential methylation using the top 200 most variable CpG sites (Figure 3). The resulting cluster was divided into 3 major leaf nodes: clusters 1, 2, and 3. Cluster 1 displayed a hypomethyation signature, and more than half of BPA-high samples were in this cluster (Figure 3A and B). While the least number of BPA-high samples were categorized into the cluster 2, it contained the greatest number of BPA-low and -intermediate samples. In general, BPA-low and -intermediate samples showed similar methylation profiles in all resulting leaves, indicating similarity in CpG methylation between these two groups. Lastly, cluster 3, which exhibited a high methylation signature was evenly composed of samples from all three groups (Figure 3). The tree plot of mean-centered β-scores of these top 200 most variable CpG sites is provided in Additional file 2: Figure S2.

An enrichment analysis on the dichotomous model candidate sites was performed using LRpath with resulting p-value output from R statistical analysis [42]. Highly enriched GO molecular functions included various receptor and receptor binding activities of G-protein-coupled receptor (p-value < 1.91e-5 and FDR < 2.00e-3), chemokine (p-value < 2.48e-5 and FDR < 2.02e-3), G-protein chemoattractant, and other sensory-related receptors (Table 1 and Additional file 1: Table S4). The cytokine-related concepts were further supported by a top-ranked related KEGG pathway concept (p-value < 3.06e-6 and FDR < 5.69e-4). Other top KEGG pathways were involved with metabolism and biosynthesis activities (Additional file 1: Table S4), and GO terms were enriched with immune and inflammatory responses. Interestingly, these concepts were enriched among the hypomethylated gene sets, indicating possible induced activity with higher BPA concentrations. The enrichment analysis using a stringent BPA-high group (N = 5), whose BPA measurements were above the mean of age-matched American girls, yielded similar findings, with immune-related and metabolic concepts enriched among hypomethylated genes.

**Table 1:** LRpath analysis reveals that cytokine and hormone activity, immune response, and metabolic processes among the top enriched concepts within differentially methylated genes

**Dichotomous model - pathway (FDR <0.2, P-value <0.02)**							
**Name**	**Concept type**	**#Genes**	**Coeff**	**Odds ratio**	**P-Value**	**FDR**	**Enriched**
Cytokine-cytokine receptor interaction	KEGG Pathway	229	−0.200253028	0.288086617	3.06E-06	5.69E-04	Down
Metabolism of xenobiotics by cytochrome P450	KEGG Pathway	61	−0.287221818	0.167801921	1.58E-04	0.014739788	Down
Vitamin A (retinol) metabolism	EHMN Pathway Gene	25	−0.428286702	0.069834019	3.14E-04	0.015401391	Down
N-Glycan biosynthesis	KEGG Pathway	44	0.359631955	9.346216996	0.001289861	0.079971357	Up
Proteogylcan biosynthesis	EHMN Pathway Gene	21	−0.40681778	0.079801493	0.001359963	0.0333191	Down
Oxidative phosphorylation	KEGG Pathway	101	0.206370232	3.605679051	0.004079267	0.176257686	Up
C21-Steroid hormone metabolism	KEGG Pathway	12	−0.42731737	0.07025597	0.00473811	0.176257686	Down
Proteasome	KEGG Pathway	21	0.442939628	15.68486443	0.006686061	0.18372292	Up
Ribosome	KEGG Pathway	59	0.255415299	4.890555948	0.006914303	0.18372292	Up
Natural killer cell mediated cytotoxicity	KEGG Pathway	116	−0.157029295	0.376862256	0.008222671	0.188057588	Down
**Dichotomous model - GO (FDR <0.01, P-value <5e-4, Odds ratio >5)**							
**Name**	**Concept type**	**#Genes**	**Coeff**	**Odds ratio**	**P-value**	**FDR**	**Enriched**
Epidermis development	GO Biological Process	120	−0.288785876	0.166178787	7.22E-08	2.83E-05	Down
Ectoderm development	GO Biological Process	130	−0.27220051	0.184220938	1.69E-07	4.41E-05	Down
Sensory perception of chemical stimulus	GO Biological Process	81	−0.326598184	0.131377584	2.59E-07	5.80E-05	Down
Digestion	GO Biological Process	85	−0.314243619	0.141861941	4.72E-07	8.22E-05	Down
Ribonucleoprotein complex biogenesis and assembly	GO Biological Process	161	0.282212002	5.776725456	5.25E-07	8.23E-05	Up
Regulation of homeostatic process	GO Biological Process	18	−0.534094322	0.03618224	2.19E-06	3.12E-04	Down
G-protein-couples receptor binding	GO Molecular Function	69	−0.298251857	0.156684914	1.91E-05	0.001995771	Down
Chemokine binding	GO Molecular Function	26	−0.431856927	0.068301636	2.48E-05	0.002021324	Down
Sensory perception of smell	GO Biological Process	56	−0.318324204	0.138309662	2.90E-05	0.002387889	Down
Serine-type endopeptidase inhibitor activity	GO Molecular Function	73	−0.285841884	0.169247136	2.90E-05	0.002021324	Down
**Continuous model - pathway (FDR <0.2, P-value <0.02)**							
**Name**	**Concept type**	**#Genes**	**Coeff**	**Odds ratio**	**P-Value**	**FDR**	**Enriched**
Cytokine-cytokine receptor interation	KEGG Pathway	229	−0.205604496	0.278663207	9.68E-09	1.80E-06	Down
Metabolism of xenobiotics by cytochrome P450	KEGG Pathway	61	−0.2927432	0.162141761	4.33E-06	4.03E-04	Down
Complement and coagulation cascades	KEGG Pathway	66	0.25256689	0.208127535	5.11E-05	0.003167145	Down
Plaminogen activating cascade	Panther Pathway	13	−0.497645229	0.045380623	6.32E-05	0.114613557	Down
Cytokines and Inflammatory Response	Biocarta Pathway	29	−0.33471697	0.124913374	195E-04	0.035217055	Down
Ribosome	KEGG Pathway	59	0.287062223	5.953499593	2.21E-04	0.010264183	Up
Vitamin A (retinol) metabolism	EHMN Pathway Gene	25	−0.385600917	0.091048898	2.51E-04	0.012317017	Down
Autoimmune thyroid disease	KEGG Pathway	47	−0.26174305	0.196590877	3.38E-04	0.012575822	Down
Oxidative phosphorylation	KEGG Pathway	101	0.209255664	3.670918657	4.30E-04	0.013316379	Up
Asthma	KEGG Pathway	26	−0.321259111	0.135809858	6.79E-04	0.015209508	Down
**Continuous model - GO (FDR <0.01, P-value <5e-4, Odds ratio >5)**							
**Name**	**Concept type**	**#Genes**	**Coeff**	**Odds ratio**	**P-Value**	**FDR**	**Enriched**
Sensory perception of chemical stimulus	GO Biological Process	81	−0.378994195	0.094865006	8.35E-14	6.54E-11	Down
Digestion	GO Biological Process	85	−0.332578826	0.126584265	6.03E-11	2.10E-08	Down
Epidermis development	GO Biological Process	120	−0.281910372	0.17343325	2.06E-10	5.38E-08	Down
Ectoderm development	GO Biological Process	130	−0.26538019	0.192197099	7.20E-10	1.34E-07	Down
Sensory perception of smell	GO Biological Process	56	−0.372825845	0.09857215	7.72E-10	1.34E-07	Down
Acute inflammatory response	GO Biological Process	69	−0.321398071	0.135692626	1.28E-08	1.55E-06	Down
Serine-type endopeptidase inhibitor activity	GO Molecular Function	73	−0.304550109	0.150670556	3.09E-08	2.71E-06	Down
olfactory receptor activity	GO Molecular Function	42	−0.377710874	0.095624611	3.89E-08	2.71E-06	Down
Immunoglobulin mediated immune response	GO Biological Process	50	−0.352071918	0.11214204	5.47E-08	5.72E-06	Down
B cell mediated immunity	GO Biological Process	51	−0.34665743	0.115979703	7.23E-08	7.08E-06	Down
**Continuous model - metabolite**							
Progesterone	Metabolite	34	−0.456431331	0.058628061	2.25E-07	1.04E-04	Down
Testerone	Metabolite	53	−0.364806957	0.103608877	5.41E-07	1.25E-04	Down
Trichloroethanol	Metabolite	46	−0.365998732	0.102844341	2.20E-06	2.59E-04	Down
Estradiol-17beta	Metabolite	47	−0.362340859	0.105208999	2.25E-06	2.59E-04	Down

This table depicts the top 10 GO and pathway concepts in both the dichotomous and continuous analyses.

As a second approach, modeling BPA as a continuous linear model utilizing all 46 samples identified 3,042 differentially methylated sites with a p-value < 0.05 and 523 sites with a p-value < 0.01 (Figure 2B and Additional file 1: Table S3). Seventy-six percent of the continuous model sites overlap with the initial categorical modeling hits. We uploaded significant CpG sites from the continuous approach to LRpath for gene set enrichment testing, and observed a high resemblance between dichotomous and continuous LRpath pathways. For example, highly enriched GO molecular functions and pathways included receptor binding activities such as G-protein, chemokine, and sensory-related, as well as immune, and inflammatory responses (Table 1 and Additional file 1: Table S4). Significantly enriched GO and pathway terms among hypermethylated sites were ribosome-related, such as structural constituent of ribosome, rRNA metabolic process, rRNA processing, and ribosome biogenesis and assembly. On the other hand, enriched concepts among hypomethylated sites included metabolite concepts such as progesterone (p-value < 2.25e-7 and FDR < 1.04e-4), testosterone (p-value < 5.41e-7 and FDR < 1.25e-4), and estradiol-17beta (p-value < 2.25e-6 and FDR < 2.59e-4). The ratio of the odds of an event was 17, 9.6, and 9.5 respectively.

### Higher BPA concentration is associated with hypomethylation of X-chromosome CpG sites

**Figure 4:**
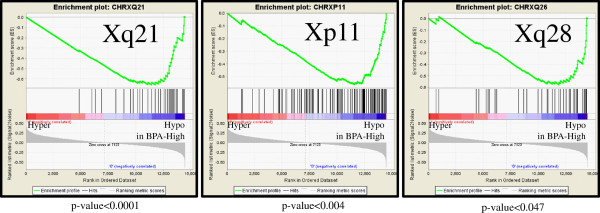
In the dichotomous model analysis, multiple regions in chromosome X were enriched with hypomethylated genes in BPA-high vs. BPA-low groups.

Using the dichotomous categorical model, we investigated the chromosomal location of the differentially methylated CpG sites. The differences in mean β-score of each probe between BPA-low and BPA-high groups were pre-ranked and subjected to the GseaPreranked analysis [46]. Several cytogenic regions on chromosome X, including both the p and q arms (Xq21, Xp11, Xq24, etc.) were significantly enriched (p-value < 0.05) with hypomethylated probes in BPA-high vs. BPA-low samples (Figure 4 and Additional file 1: Table S5). No regions from chromosome X were enriched with hypermethylated genes in BPA-high vs. BPA-low groups, and none of the autosomes were significantly enriched in either direction. Chromosome X enrichment is also observed, even when BPA-intermediate group is added to the analysis and combined with the BPA-low group (data not shown). However, when the BPA-intermediate group is combined with BPA-high group to be tested against BPA-low group, the enrichment within chromosome X is no longer significant.

### Imprinted gene methylation is not broadly affected by BPA concentrations

The methylation patterns observed in differentially methylated regions (DMRs) of imprinted regions in the human genome play an important role in parent-of-origin monoallelic gene expression. We compared the methylation level of imprinted genes obtained from the catalogue of parent-of-origin effects (http://igc.otago.ac.nz/home.html). The methylation differences of 34 unique imprinted genes measured by 233 probes on BeadChip in BPA-low and BPA-high samples were evaluated, and none were found to be differentially methylated by BPA exposure group (Additional file 2: Figure S3A) among Egyptian pre-adolescent girls, with the exception of Necdin (*NDN*). Among six probes (999, 776, 80, and 77 bps downstream, and 308 and 170 bps upstream of TSS) targeting the promoter of *NDN*, one CpG site (308 bp upstream from TSS) was significantly less methylated in BPA-high samples (p-value < 0.02) with average methylation difference greater than 5% (Additional file 2: Figure S3B). Neighboring CpG sites (77 bp downstream and 170 bp upstream of TSS) were not differentially methylated.

### Differential methylation is associated with known BPA-interaction genes

**Figure 5:**
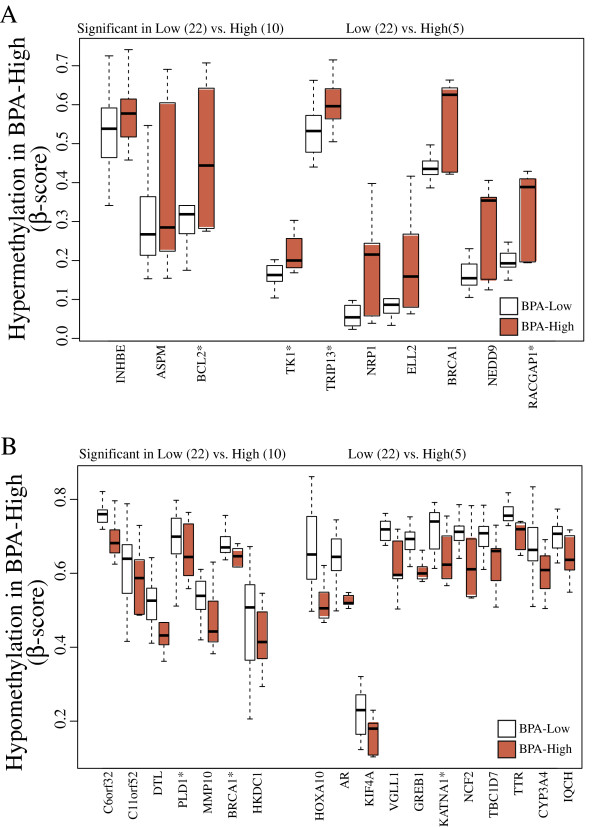
**Differential methylation observed in genes known to be interacting with BPA from the Comparative Toxicogenomics Database (CTD).** (**A**) Top candidate genes that undergo hypermethylation in BPA-high vs. BPA-low groups. (**B**) Top candidate genes that undergo hypomethylation in BPA-high vs. BPA-low groups. The reported expression change in genes with the asterisk did not inversely correlate with the methylation status.

The publicly available Comparative Toxicogenomics Database (CTD) provides manually curated information on environmental chemicals, interacting genes, and associated health effects in human as well as animal models [31-33]. A list of 903 genes in humans whose expression levels are known to be affected by BPA was acquired using a chemical-gene interaction query, yielding 333 genes with increased and 257 genes with decreased level of expression upon BPA exposure. The directionality of the expression changes in remaining 313 genes was not available. A total of 657 unique genes (from 1,355 probes) from this list were represented on the BeadChip, and the methylation levels were compared to identify “biologically-prioritized” candidate genes with previously characterized altered expression by the CTD. Overall, 293 out of 1,355 sites (21.6%) were differentially methylated (p-value < 0.05) with BPA exposure in either the dichotomous or continuous model described in the Methods. The top biologically-prioritized candidates with an average β-score difference more than 5% are shown in Figure 5 and Additional file 1: Table S6 (n = 35). In general, hypomethylated candidates exhibit high level β-scores (above 0.5), while hypermethylated candidates tend to have basal or low level methylation (Figure 5).

Several hyper- and hypo-methylated genes in BPA-high compared to BPA-low samples corresponded with expression change, adhering to the general agreement that methylation and expression often exhibit inverse directionality. For example, the hypomethylated probes mapping to C11orf52, *HOXA10*, *KIF4A*, *DTL*, *GREB1,* and *NCF2* were identified in CTD to have increased expression, while the hypermethylated probes mapping to *NCK1*, *CSDA*, and *BRCA1* were reported by CTD to have decreased expression with BPA exposure. The directionality of the expression change in hypomethylated genes including *C6orf32, MMP10*, *HKDC1*, *SLC3A1*, *VGLL1*, and *IQCH*, and hypermethylated genes including *INHBE*, *ASPM*, *NRP1*, *ELL2*, and *NEDD9* was not provided in the CTD. The expression change of the hypomethylated genes, *PLD1*, *AR*, *KATNA1*, and *CYP3A4*, and the hypermethylated genes, *BCL2*, *TK1*, *TRIP13*, and *RACGAP1*, was not inversely correlated with the methylation status.

### Candidate gene validation

The high ranking CpG sites from four candidate genes, characterized as either A) agnostic based on p-value alone (*BEX2* and *cXorf23;* Additional file 1: Table S3) or B) biologically prioritized based on a previously established association with BPA (*HOXA10* and *DTL;* Additional file 1: Table S6) were validated by quantitative bisulfite pyrosequencing (Additional file 1: Table S2). *DTL* with two CpG sites and *HOXA10* with a single CpG site were run in duplicate on all samples with sufficient DNA, resulting in high correlation between replicates (N = 59, including the original 46 profiled on the BeadChip). The regions encoding *BEX2* with 3 CpG sites and *cXorf23* with a single CpG site were each run in a single sample well due to DNA quantity limitations (N = 59 including the original 46 profiled on BeadChip).

**Figure 6:**
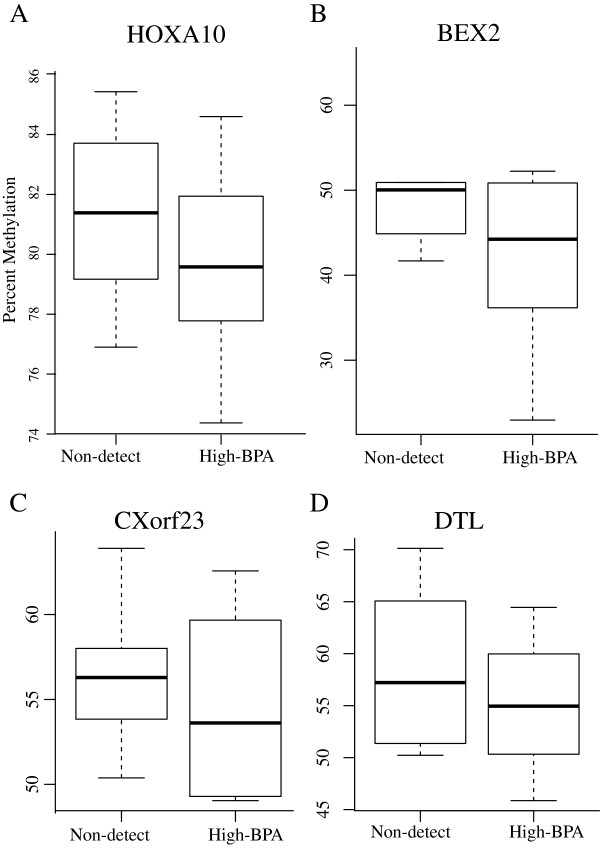
**Quantitative levels of methylation for *HOXA10*, *BEX2*, *CXorf23*, and *DTL *(N = 12 for Non-detect and 13 for High-BPA for *HOXA10*, *CXorf23*, and *DTL*, and N = 9 for Non-detect and N = 9 for High-BPA for *BEX2*).**

When methylation levels were compared between girls classified as BPA-low (<0.7 ng/mL, N = 22) to girls classified as BPA-high (>2 ng/mL, N = 13), no significant differences were observed at either the agnostic or biologically prioritized CpG sites (data not shown). However, when the analysis was restricted to girls with urinary concentrations of BPA below the limit of detection (N = 12) compared to girls characterized as BPA-high (N = 13), a decreasing, yet statistically non-significant, trend in DNA methylation in all candidate CpG sites was observed in all 4 genes (Figure 6A-D). Interestingly, further dichotomization of exposure categorization to girls with non-detectable concentrations of BPA (N = 12) and those with BPA concentrations 2 s.d. above the mean (>4 ng/mL, N = 3) revealed differential methylation with statistical significance (p-value < 0.024) at the *HOXA10* biologically-prioritized candidate gene (Additional file 2: Figure S4).

## Discussion

The effect of environmental exposures on epigenetic regulation in human populations remains poorly understood. The consequences of maternal exposure to EACs, including BPA, on the epigenetic landscape of offspring has been studied using animal models, and this includes the discovery of altered DNA methylation at candidate metastable epialleles such as CDK5 activator-binding protein (*Cabp*) and viable yellow Agouti (*A*^*vy*^) alleles [17,19]. The effects of postnatal BPA exposure on the human epigenome, however, remain largely unexplored. To date, the majority of environmental epidemiology studies that incorporate epigenetics have been conducted in the context of a cohort study, where both environmental exposures and epigenetic outcomes are quantified in healthy populations [47-49]. The majority of these studies have looked at single genes or global methylation profiles. Increasingly, epigenome-wide approaches are useful in identifying a broader constellation of epigenetic changes associated with environmental exposures [50]. Here we measured DNA methylation levels genome-wide in 46 pre-adolescent Egyptian girls with varying concentrations of BPA exposure. The study population was homogeneous with respect to age and genetic population stratification was limited, thereby increasing our ability to identify exposure based methylation profiles.

While the overall levels of urinary BPA concentrations of Egyptian girls were low and less variable compared to age-matched American girls [27], we identified several CpG sites showing differential methylation upon exposure and biological pathways of interest using a genome-wide approach. This provides support for the hypothesis that even low concentrations of BPA exposure may be associated with health effects via epigenetic modification, and studies of relevant ranges of BPA exposure in human populations are warranted. These results should be interpreted cautiously as the use of spot urine for measuring BPA exposure is a limitation of our study. For example, interpersonal variation in amount of water consumed may affect urine output. Thus, we adjusted urine BPA concentrations using specific gravity. Similarly, to reduce variability arising from exposures throughout the day, the participants provided spot urine sample during a specified time period between noon to 4 pm. Additionally, previous studies on various populations have shown that individuals are exposed to mixtures of chemicals, potentially with similar mechanisms of action as BPA, such as phthalates, pesticides, and heavy metals. Future studies using more comprehensive methods of exposure assessment, including the measurement of chemical mixtures, are warranted [51].

Of interest, many previously identified pathways associated with BPA exposure were also identified in our analysis as top differentially methylated pathways. LRpath enrichment analysis identified BPA-induced hypomethylation among genes involved in metabolism and steroid hormones, including progesterone, testosterone, and estradiol. BPA is one of several widely known EACs or xenoestrogens [52,53], and steroid hormones are known to influence metabolism and inflammation including various immune responses. As such, several immune-response concepts were enriched with hypomethylated CpG sites in girls in the BPA-high group. In previous studies, higher cytokine activity was observed in BPA-exposed rodents [54]. In addition, the autoimmune thyroid disease pathway was significantly enriched with hypomethylated genes in girls categorized in the BPA-high group, and BPA is a known thyroid receptor antagonist [55,56]. Genes involved in the N-glycan biosynthesis pathway were also found to be differentially methylated based on BPA exposure, supporting previous work linking BPA concentrations with insulin resistance and type II diabetes [57], For example, N-glycans are key players mediating cell-to-cell communication, interacting with glycan-binding proteins [58], and can be attached to human insulin receptor (IR) and interfere with the IR interaction with antibodies or binding proteins by masking the surface of IR dimers [59].

Prenatal BPA exposure has been associated with altered DNA methylation and expression of imprinted genes in mouse embryos [60,61]. A methylation analysis of oocytes from CD-1 mice perinatally exposed to 20 or 40 ug BPA/kg diet identified decreased methylation among imprinted genes, including insulin like growth factor 2 receptor (*IGF2R*) and paternally expressed 3 (*PEG3*) [60]. When pregnant JF1 mice were exposed to 200 ug BPA/kg diet, embryos showed a 10% difference in *Slc22a18* expression compared with controls [61]. When we examined DNA methylation of 34 imprinted genes (from 233 sites) in these subjects, BPA-associated changes were not observed in the imprinted genes, with the exception of a single CpG site in the *NDN* promoter (Additional file 2: Figure S3B). The imprinted gene, *NDN*, is located within the Prader-Willi/Angelman syndrome (PWS) chromosomal region [62]. Loss of function of this gene has been associated with post-natal lethality [63]; thus *NDN* has a critical role during development. Thus, methylation profiles at imprinted genes, while labile to nutritional and environmental influences early in development [64-66], may remain stable and relatively resistant to environmental factors over the life course.

DNA methylation of CpG islands acting in concert with other epigenetic mechanisms, are known to be important in female mammalian X chromosome inactivation and maintenance [67]. As with genomic imprinting, chromosome X inactivation may thus be potentially vulnerable to the effects of environmentally-induced epigenetic reprogramming [68]. In our data, we observed an enrichment of hypomethylated sites on chromosome X with increasing BPA concentrations (Figure 4 and Additional file 1: Table S5). One of the significantly hypomethylated cytobands in chromosome X was Xq13 (p-value < 0.02), where the X Inactivation Center (XIC) is located. The XIC is known to play a role in *Xist* expression, a major regulator of X inactivation. Two of the four sites validated by quantitative bisulfite pyrosequencing were *CXorf23* (Xp22.12) and *BEX2* (Xq22.1) from chromosome X. Although we were unable to achieve robust statistical significance as seen in the BeadChip data, we did observe a slight decrease in DNA methylation in BPA-high samples (Figure 6). As the girls observed in our study had low urinary BPA concentrations compared to age-matched American girls [27], it will be important to further evaluate the chromosome X findings in other exposed populations.

A functional validation of DNA methylation targets using gene expression was not performed in this cohort due to the unavailability of field-collected RNA. To overcome this limitation, we extracted gene expression information from multiple previously conducted studies available in CTD. The increasing wealth of data in the public arena now allows for the comparison of our genome-wide signatures with those from other studies, efficiently providing additional insight into key biologically-relevant pathways. When the curated genes known to interact or harbor differential expression upon BPA exposure in the CTD were compared with the list of differentially methylated genes in our study, more than 20% of BPA-associated genes were found to overlap. The biologically-prioritized candidate gene, *BEX2*, is associated with estrogen, cell cycle, and apoptosis pathways [69] and over-expressed in Ishikawa cells, a well-differentiated human endometrial adenocarcinoma cells, treated with BPA [70]. In addition, the biologically-prioritized candidate, *HOXA10*, has previously been associated with decreased DNA methylation among *in utero* BPA-exposed mouse samples [71]. Altered methylation at *HOXA10* was confirmed by bisulfite pyrosequencing in our Egyptian girls, despite relatively low BPA concentrations with small variation compared to US populations [27]. Although the validation did not result in the same depth of significance as seen in the array data, it showed a clear trend toward differential methylation (Additional file 2: Figure S4). Importantly, some biologically-prioritized candidate genes are known to target the major disease pathways, such as DNA repair, cell cycle, and development. Thus, future epigenetic epidemiology studies with increased sample size and increased exposure ranges are needed to further elucidate the consequences of genome-wide altered methylation upon BPA exposure, including *BRCA1*, *BEX2*, and *HOXA10*.

## Conclusions

BPA exposure levels in Egyptian pre-adolescent girls were relatively low with less variability compared to age-matched American girls; however, using a genome-wide methylation approach, we identified alterations in saliva DNA methylation associated with BPA concentrations. Moreover, a number of identified genes are important regulators in developmental processes and/or disease susceptibility. Pathways involved in metabolism, steroid hormone, thyroid disease, and immune response were also enriched among differentially methylated genes. The integrative analysis with data obtained from the CTD database identified candidate genes, where epigenetic modifications may be associated with concomitant transcriptomic changes observed upon BPA exposure. Although methylation is more susceptible to environmental exposures during early development, these data indicate that BPA may affect human health through specific epigenomic modification of genes in relevant pathways even throughout pre-adolescent development.

## Abbreviations

Avy: Viable yellow agouti; BPA: Bisphenol A; BMI: Body mass index; Cabp: CDK5 activator-binding protein; CTD: Comparative Toxicogenomics Database; DES: Diethylstilbestrol; DMRs: Differentially methylated regions; DOHaD: Developmental origins of health and disease; EACs: Endocrine-active compounds; EHMN: Edinburgh Human Metabolic Network; GO: Gene ontology; GSEA: Gene set enrichment analysis; IGF2: Insuline-like growth factor 2; IGF2R: Insulin like growth factor 2 receptor; IR: Insulin receptor; KEGG: Kyoto Encyclopedia of Genes and Genomes; LIMMA: Linear models for microarray data; LOD: Limit of detection; LRpath: Logistic regression based pathway analysis tool; NDN: Necdin; NHANES: National Health and Nutrition Examination Survey; Pde4d4: Phosphodiesterase type 4 variant 4; PEG3: Paternally expressed 3; PWS: Prader-Willi/Angelman Syndrome; RPMM: Recursively partitioned mixture model.

## Competing interests

The authors declare that they have no competing interests.

## Authors’ contributions

AH, ASS, DCD, IAS, and LSR conceived of this project and designed the study protocols. AH, ASS, and IAS supervised and conducted field collection, including subject recruitment. AMC was responsible for laboratory analysis of BPA. JHK, MSN, and MAS performed statistical analysis. JHK, JAC, and CW carried out pyrosequencing analysis. JHK, DCD, and LSR drafted the manuscript, and all authors edited and approved the final draft of the manuscript.

## Supplementary Material

Additional file 1: Table S1 Distribution of urinary BPA concentration (ng/ml). **Table S2.** Pyrosequencing Primer sequences. **S3.** Candidate Genes. **Table S4.** Gene ontology enrichment analysis using LRpath. **Table S5.** Cytoband enrichment analysis using GSEA. **Table S6.** Differential methylation in BPA interacting genes. Click here for file

Additional file 2: Figure S1 Average percent change in differentially methylated probes (p-value < 0.05) between BPA-low and BPA-high samples (N=1,439). **Figure S2.** The tree plot of mean-centered β-scores of top 200 most variable CpG sites. **Figure S3.** The levels of methylation observed among imprinted genes in Egyptian cohort (A) Averaged β-score of 34 unique genes from 233 probes is displayed in heatmap. Red color indicates higher levels of methylation. (B) The β-score boxplots of a CpG site located 308 bp upstream of *NDN* promoter from BPA-Low and BPA-High groups. **Figure S4.** Quantitative levels of *HOXA10* methylation in the Egyptian girls cohort. Click here for file
