# Supplementary material for: Bisphenol A-associated epigenomic changes in prepubescent girls: a cross-sectional study in Gharbiah, Egypt
Source: Environ Health. 2013 Apr 16;12:33. doi: 10.1186/1476-069X-12-33 (PMC3655072; doi:10.1186/1476-069X-12-33)
Supplement: Additional file 2: Figure S1 — Average percent change in differentially methylated probes (p-value < 0.05) between BPA-low and BPA-high samples (N=1,439). Figure S2. The tree plot of mean-centered β-scores of top 200 most variable CpG sites. Figure S3. The levels of methylation observed among imprinted genes in Egyptian cohort (A) Averaged β-score of 34 unique genes from 233 probes is displayed in heatmap. Red color indicates higher levels of methylation. (B) The β-score boxplots of a CpG site located 308 bp upstream of NDN promoter from BPA-Low and BPA-High groups. Figure S4. Quantitative levels of HOXA10 methylation in the Egyptian girls cohort. [file 1476-069X-12-33-S2.docx]

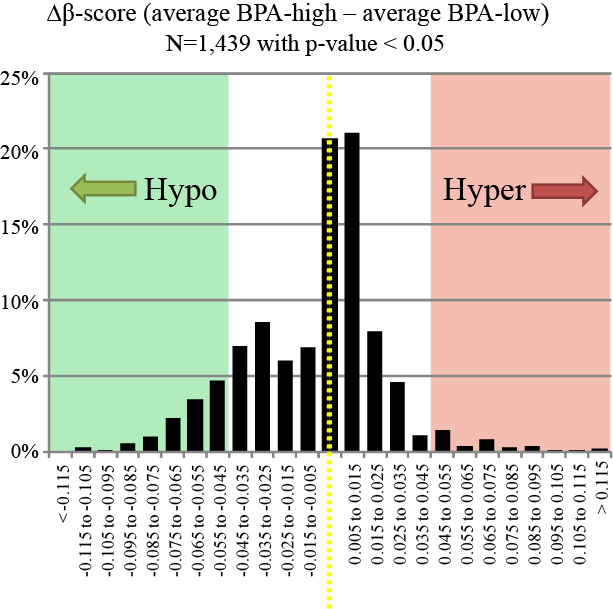


Supplementary Figure 1: Average percent change in differentially methylated probes (p-value < 0.05) between BPA-low and BPA-high samples (N=1,439).


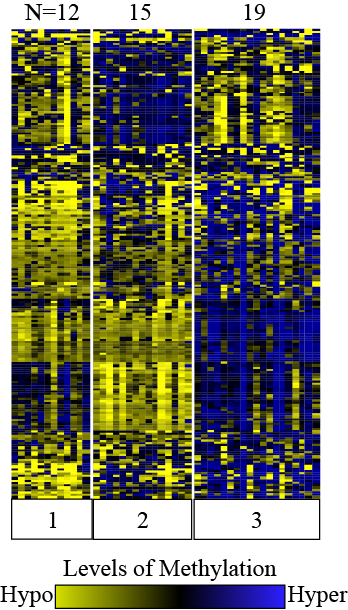


Supplementary Figure 2: The tree plot of mean-centered β-scores of top 200 most variable CpG sites.


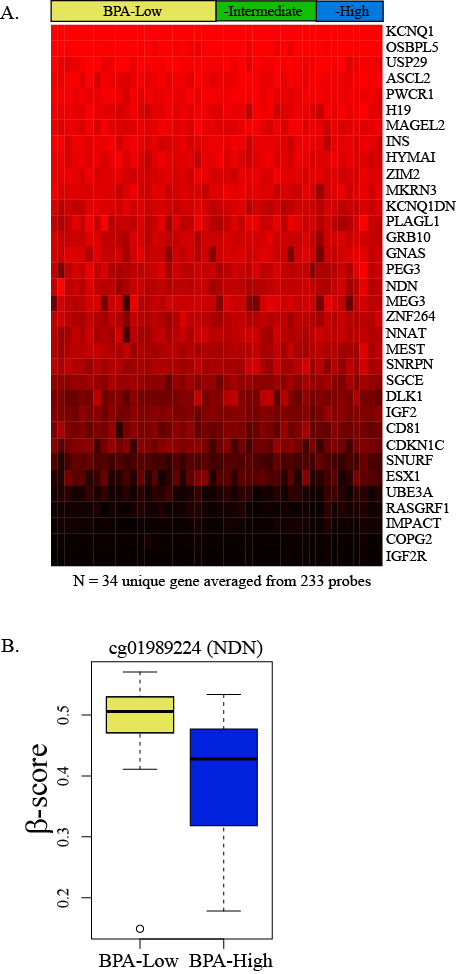


Supplementary Figure 3: The levels of methylation observed among imprinted genes in Egyptian cohort (A) Averaged β-score of 34 unique genes from 233 probes is displayed in heatmap. Red color indicates higher levels of methylation. (B) The β-score boxplots of a CpG site located 308 bp upstream of *NDN* promoter from BPA-Low and BPA-High groups.


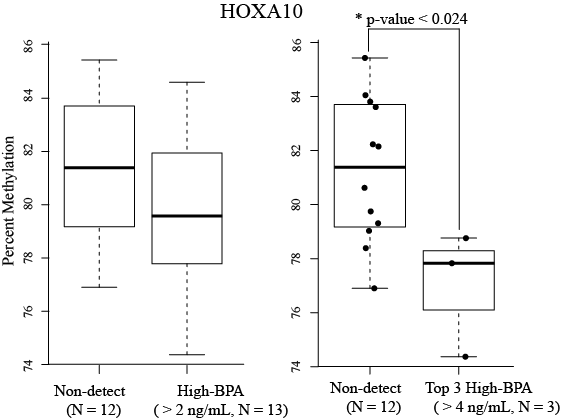


Supplementary Figure 4: Quantitative levels of *HOXA10* methylation in the Egyptian girls cohort.
